# A new marine-derived sulfoglycolipid triggers dendritic cell activation and immune adjuvant response

**DOI:** 10.1038/s41598-017-05969-8

**Published:** 2017-07-24

**Authors:** Emiliano Manzo, Adele Cutignano, Dario Pagano, Carmela Gallo, Giusi Barra, Genoveffa Nuzzo, Clementina Sansone, Adrianna Ianora, Konrad Urbanek, Daniela Fenoglio, Francesca Ferrera, Cinzia Bernardi, Alessia Parodi, Giuseppe Pasquale, Antonio Leonardi, Gilberto Filaci, Raffaele De Palma, Angelo Fontana

**Affiliations:** 1 Bio-Organic Chemistry Unit, CNR- Institute of Biomolecular Chemistry, Via Campi Flegrei 34, IT-80078 Pozzuoli, Napoli, Italy; 2 University of Campania, Clinical Immunology and Allergology, Dept. of Internal and Experimental Clinic, c/o II Policlinico (Bd. 3), Via S. Pansini, 5, 80131 Napoli, Italy; 3 Stazione Zoologica “A. Dohrn”, Villa Comunale, 80121 Napoli, Italy; 4 University of Campania, Dept. of Experimental Medicine, c/o II Policlinico (Bd. 3), Via S. Pansini, 5, 80131 Napoli, Italy; 5 Centre of Excellence for Biomedical Research, University of Genoa, Genoa, Italy; 6 Department of Internal Medicine, University of Genoa, Genoa, Italy; 7 Univeristy of Naples “Federico II”, Department of Molecular Medicine and Medical Biotechnology, c/o II Policlinico (Bd. 3), Via S. Pansini, 5, 80131 Napoli, Italy; 8 Institute of Protein Biochemistry, via P. Castellino, 111, 80131 Napoli, Italy

## Abstract

Dendritic Cells (DCs) recognize infectious non-self molecules and engage the adaptive immune system thereby initiating long lasting, antigen-specific responses. As such, the ability to activate DCs is considered a key tool to enhance the efficacy and quality of vaccination. Here we report a novel immunomodulatory sulfolipid named β-SQDG18 that prototypes a class of natural-derived glycolipids able to prime human DCs by a TLR2/TLR4-independent mechanism and trigger an efficient immune response *in vivo*. β-SQDG18 induces maturation of DC with the upregulation of MHC II molecules and co-stimulatory proteins (CD83, CD86), as well as pro-inflammatory cytokines (IL-12 and INF-γ). Mice immunized with OVA associated to β-SQDG18 (1:500) produced a titer of anti-OVA Ig comparable to traditional adjuvants. In an experimental model of melanoma, vaccination of C57BL/6 mice with β-SQDG18-adjuvanted hgp10 peptide elicited a protective response with a reduction in tumour growth and increase in survival.

## Introduction

**Figure 1:**
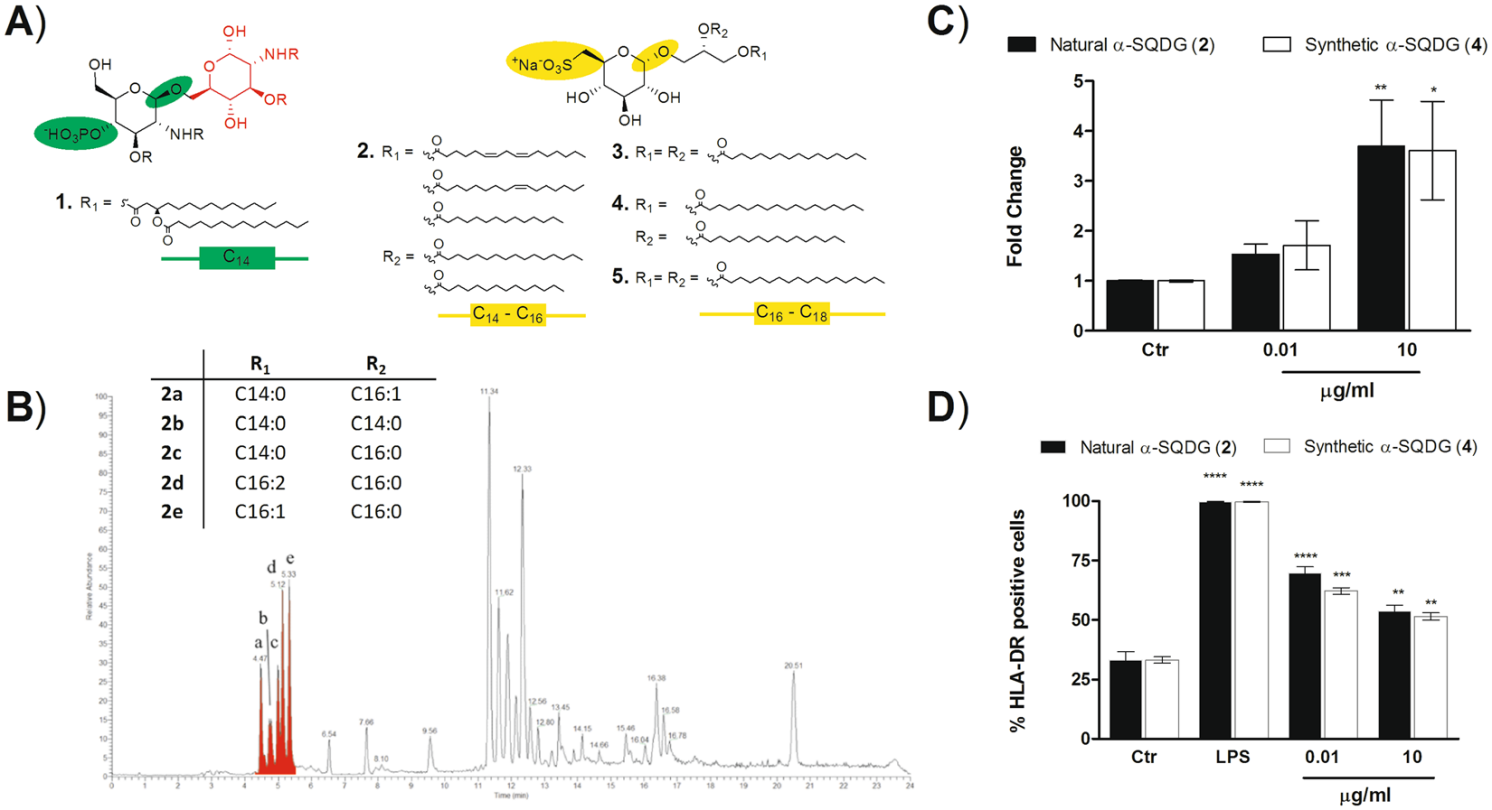
Chemical structure and DC activation of natural sulfoquinovosides (α-SQDG) from marine diatoms. (**A**) Structures of DC agonists: monophosphoryl lipid A (MPLA, **1**) (reference compound), natural α-SQDG (**2**) and synthetic (**3**-**5**) α-SQDG. Colors underline the chemical differences in type and position of the anionic function, configuration of the anomeric carbon, second sugar residue and fatty acid acyl chain between MPLA (**1**) and α-SQDG (**2**-**5**); (**B**) LC-MS profile of active α-SQDG (**2**) in the extract of the marine diatom *Thalassiosira weissflogii*. The insert reports the type and distribution of fatty acids bound to the sulfoquinovosyl glycerol residue; (**C**) expression of IL-12 (mean/standard deviation) and (**D**) expression of HLA-DR by MoDCs stimulated with natural and synthetic analogs of α-SQDG at 10 ng/mL and 10 µg/mL. Results (mean +/− standard deviation) are reported as percentage of positive cells (marker expression value over 10^4^) out of the total DC population. LPS was used as positive control. Asterisks indicate significant differences from the cells treated only with vehicle (control, Ctr) at a 95% (P < 0.05) confidence level, as determined using two-way ANOVA analysis.

Stimulation of an immune response by using attenuated or inactivated biological agents has been the traditional basis for vaccination against viral and bacterial infections. However, most of the recent vaccines are comprised of highly purified synthetic macromolecules, such as peptides or recombinant DNA produced by genetic engineering technology. These antigens tend to be safer but poorly immunogenic; therefore they need to be combined with agents known as adjuvants that potentiate the immune response mostly by activation of specific accessory cells, named Antigen-Presenting Cells (APCs)^1–6^. Adjuvants are a highly heterogeneous group of compounds with a common property¸ usually defined as adjuvancy, to enhance the immunogenicity of antigens. Nevertheless, except for the Toll-like receptor (TLR) agonist monophosphoryl lipid A (MPLA, compound 1 in Fig. 1), clinically approved formulations are restricted to aluminium salts and emulsions of lipids (e.g. squalene) in water^7^. Dendritic cells (DCs) are the most efficient APCs^8–14^ and are often called ‘nature’s adjuvant’^15^ for their ability to induce activation and specific expansion of CD4^+^ helper T (Th) and CD8^+^ cytotoxic T (CTL) lymphocytes determining the functional profile of these cells against bacterial and viral antigens^15^. For these reasons DCs have become the product of choice for the preparation of DC-based vaccines against tumors or infections^16–19^.

Here we provide the first evidence of a novel class of molecular adjuvants of marine origin able to stimulate *in vitro* DC maturation and prime *in vivo* specific immune response.

## Results

### Response of Dendritic cells to natural marine lipids

DCs are heterogeneous and derive from progenitors in the bone marrow from where they migrate to all tissues in the body. Monocytes are also able to differentiate into dendritic cells (MoDCs) when subjected to an inflammatory stimulus; these cells are able to preserve the main characteristics of normal APCs^10, 20^. Fully differentiated MoDCs efficiently present soluble and cell-associated antigens to T cells and show stimulating capacity comparable to tissue-derived DCs^21^. In this study, human MoDCs were placed in 96 multiwell plates and treated with methanol extracts from a collection of marine organisms including invertebrates and photosynthetic protists (microalgae, diatoms and dinoflagellates). Evolution from immature antigen-capturing cells to mature, immunologically competent DCs was assessed by measuring the expression of MHC-Class II receptor HLA-DR and the co-stimulatory molecule CD86 by flow cytometry^22^. When mature DCs were detected, we also measured production of IL-12, a specific mediator of Th1/CTL activation^23–25^, by quantitative PCR. Extracts from different diatom species gave positive hits with the major activity associated to fractions containing glycolipids. One of the most active species, namely *Thalassiosira weissflogii* (CCMP-1336), was mass cultivated (300 g dry weight) in 70 L photobioreactors. Extraction by SPE and bioassay-guided fractionation led to the isolation of a mixture of *α*-sulfoquinovosyl diacylglycerols (*α*-SQDGs, **2**) that induced overexpression of IL-12 at 10 µg/mL and HLA-DR at 10 ng/mL (Fig. 1). *α*-SQDGs are relatively minor lipids compared to galactosylglycerides in plants but their content is usually higher in algae and microalgae. They are concentrated in the inner membranes of chloroplasts but their function is still unclear. Instead of the phosphate group of MPLA, *α*-SQDGs are characterized by a sulfur atom directly linked to the carbon 6′ of a single unit of monosaccharide referred to as sulfoquinovose (Fig. 1). Furthermore, compared to immunogenic bacterial glycolipids that usually have a β-configuration of anomeric carbons, *α*-SQDGs have an *α*-configuration of the sugar unit. Lipid profiling by LC-MS^26^ of *T. weissflogii* revealed that the natural active fraction was composed of a mixture of *α*-SQDGs differing in the composition and position of saturated or monounsaturated fatty acids, mainly palmitic (16:0) and palmitoleic (16:1) acids (Fig. 1). The immunostimulatory activity of this class of lipids was further confirmed by the effect of a few synthetic analogs (**3**-**5**)^27^ that were all able to elicit maturation and IL-12 production of DCs regardless of the presence of C_16_ or C_18_ alkyl chains.

### Activation of DC by the synthetic sulfolipid β-SQDG18

**Figure 2:**
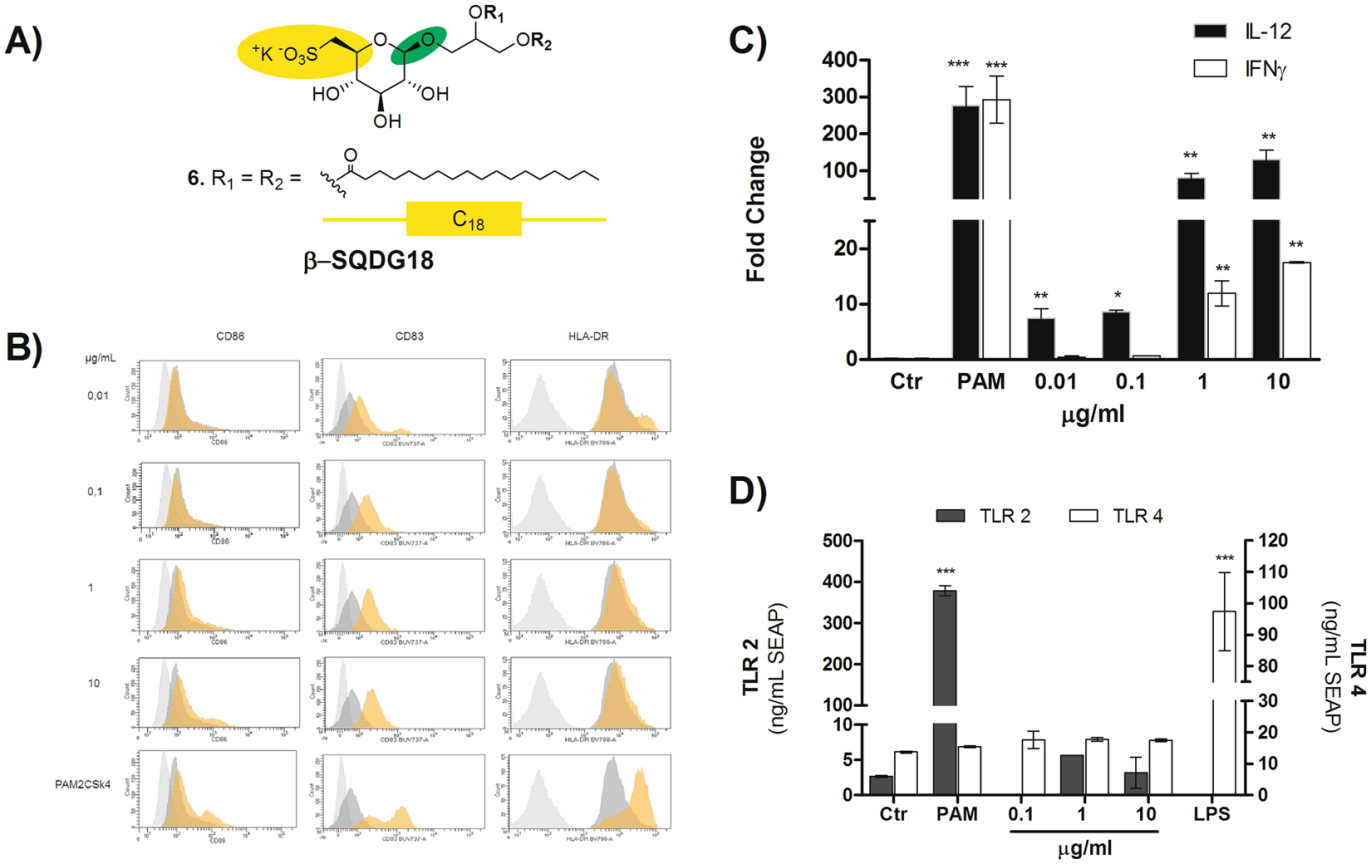
Dose-dependent priming of DC by βSQDG18 (6). (**A**) The chimeric molecule β-SQDG18 (**6**) derives from combination of the structural characteristics of α-SQDG (**yellow**) and MPLA (**green**); (**B**) Flow-cytometry analysis of the phenotyping markers of DC maturation (HLA-DR, CD83 and CD86) at concentrations between 10 ng/ml to 10 µg/ml of β-SQDG18 (**6**). gray = isotype control; dark gray = unstimulated cells; orange = stimulated cells. Pam2CSK4 (PAM) was used as positive control; (**C**) expression of IL-12 and IFN-γ by DC stimulated with β-SQDG18 in the same range of concentrations used for the above marker phenotyping; (**D**) evaluation of the Toll-Like Receptor (TLR) activation by increasing concentration concentrations of β-SQDG18 in TLR-2 and TLR-4 NF-kB/SEAP reporter cell lines. Pam2CSK4 (PAM) and LPS were used as positive controls for TLR-2 and TLR-4 respectively. Asterisks indicate significant differences from the cells treated only with vehicle (control, Ctr) at a 95% (P < 0.05) confidence level, as determined using two-way ANOVA analysis.

Inspired by the structure of MPLA (**1**), we also designed a chimeric molecule, named β-SQDG18 (compound **6** in Fig. 2), that combined the presence of the sulfonic residue typical of *α*-SQDGs with the β-configuration of the glycosidic bond that is common in immunogenic LPS-related glycolipids^28^. The chimeric sulfolipid (**6**) was synthesized by coupling 1,2-*O*-isopropylidene glycerol with the trichloroacetamidate derivative of tetra-acetyl glucose (Supplementary Information)^29^. After selective de-protection of the primary hydroxyl group at C-6′, the carbon-sulfur bond was introduced through a thioacetate intermediate from which 1,2-distearoyl-3-*O*-(β-sulfoquinovosyl)-*sn*-glycerol (β-SQDG18, **6**) was obtained by conventional chemical reactions. In comparison with natural sulfolipids, β-SQDG18 (**6**) showed an increased potency in promoting maturation of MoDCs as proved by the up-regulation of phenotypic markers in a dose-dependent manner from 10 ng/mL to 10 µg/mL. As reported in Fig. 2, the molecule increased the percentage of CD83-positive cells. High expression of this marker on the cell surface is a peculiarity of fully matured human DC^30, 31^ and critical for the *in vivo* priming of naïve T cells^32, 33^. Furthermore, upregulation of CD83 prevents MHC II and CD86 down-regulation induced by IL-10 through MARCH1 thus enhancing the immunogenicity of vaccine adjuvants^34^. Expression of CD86 by β-SQDG18 (**6**) was quantitatively not different from that induced by the TLR agonist Pam2CSK4 although the two molecules showed different ability to promote surface display of HLA-DR. β-SQDG18 (**6**) induced only a slight increase of MHC class II molecules that are﻿ constitutively expressed at high ﻿levels ﻿in DCs. The synthetic sulfolipid also stimulated production of a distinct array of pro-inflammatory cytokines including IL-12 (about 130-fold increase compared to control DMSO and about 25-fold increase compared to natural α-SQDG) and INF-γ, that are related to the DCs’ ability to prime an efficient Th1 cell response^23, 35^. In addition to these results, PCR array analysis of 96 genes in DCs stimulated by β-SQDG18 (**6**) showed an up-regulation of CD40 ligand, IL-27 and other interleukins (e.g. IL-1α and IL-1β, IL-18) that work synergistically to induce Th1 cells and play a major role in resistance to bacterial and viral infections, as well as to tumours (Supplementary Material).

### β-SQDG18 does not activate TLR2 and TLR4 response

The immune effect of MPLA (**1**) and many glycolipids of interest as vaccine adjuvants (e.g. lipoteichoic acid) is mediated by Toll-like receptors (TLRs), mainly TLR-2 or TLR-4^36^. TLRs are principal membrane-associated innate sensors that DCs and other APCs use to recognize conserved pathogen-associated molecular patterns (PAMPs). To verify whether β-SQDG18 (**6**) activity were TLR-2/TLR-4 dependent, we tested β-SQDG18 (**6**) effects in a cell-based assay with HEK293 stably co-transfected to express full-length human TLR-2 or TLR-4 and the secreted alkaline phosphatase (SEAP) reporter gene under the transcriptional control of a NF-kB response element^37^. As shown in Fig. 2D, the sulfolipid had no effect on reporter activation of either receptor at concentrations that stimulate maturation and cytokine production by DCs. Down-regulation on TLR-4 in an array analysis of 84 genes of DCs stimulated by β-SQDG18 (**6**) further supported a TLR-independent mechanism for the activation of these cells (Supplementary Material).

### Immunization of mice against ovalbumin using β-SQDG18 as adjuvant

**Figure 3:**
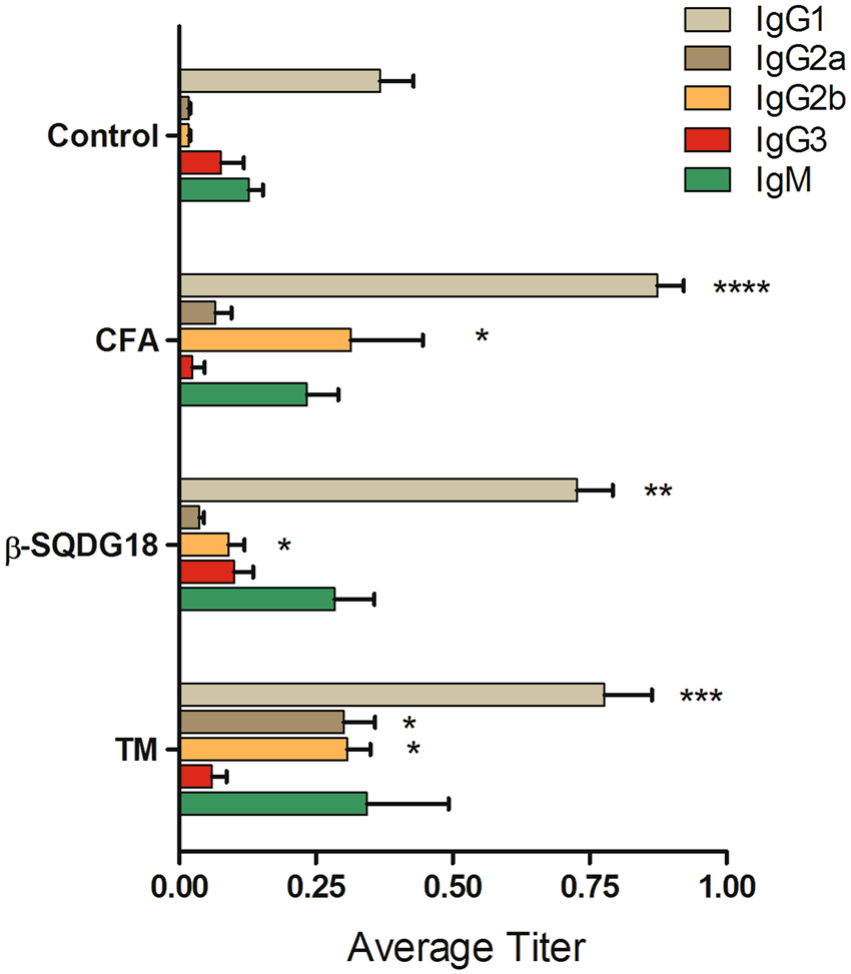
Humoral response of mice after immunization to ovalbumin formulated with β-SQDG18. C56 BL/6 mice were divided into 4 groups and inoculated twice with 5 µg antigen (OVA) in presence or absence (Control) of 2.5 mg β-SQDG18 (1:500 w/w). Co-administrations of OVA together with Complete Freund’s Adjuvant (FCA) or TiterMax (TM) were used as positive controls. Serum anti-OVA antibodies were measured by ELISA and results are shown as endpoint titer (mean/standard error). No increase in OVA-specific IgA was observed. Asterisks indicate significant differences from the control group at a 95% (P < 0.05) confidence level, as determined using two-way ANOVA analysis.

In order to acquire conclusive evidence about the potential of the synthetic sulfolipid to boost immune protection and to act as an adjuvant, the synthetic lipid was tested in animal models. First, we evaluated the antibody response in mice following immunization with OVA, as model antigen, associated to β-SQDG18 (**6**). To this aim, a sterile saline solution was formulated by mixing the protein with the synthetic sulfolipid **6** obtained by scale-up of the chemical procedure discussed above. Control groups received OVA delivered in DMSO or formulated with either complete Freund’s adjuvant (CFA) or TiterMax® (TM). Following subcutaneous injections of 5 µg OVA and 2.5 mg β-SQDG18 at day 1 and 8, the animals responded by day 21 with a robust enhancement of anti-OVA antibody titer (Fig. 3). In analogy with the control adjuvants CFA and TM, immunization with OVA/β-SQDG18 evoked increase of IgG1 and IgM. There is no univocal understanding of antibody function in mouse; however, there is good agreement about the role of IgM in early response, as well as of IgG1 in mitigation of the inflammatory process. In addition to IgG1, murine IgGs include IgG2a, IgG2b and IgG3, whose production is stimulated to different extents by the sulfolipid. Recently Collins has discussed the model of murine IgG response by concluding that these four classes of antibodies work synergistically and their actions have to be considered together^38^. In this view, the humoral response due to OVA/β-SQDG18 was comparable to the antibody titer associated with co-administration of OVA with the known adjuvants CFA or TM.

### Comparative analysis of adjuvants in vaccines against an experimental melanoma model

**Figure 4:**
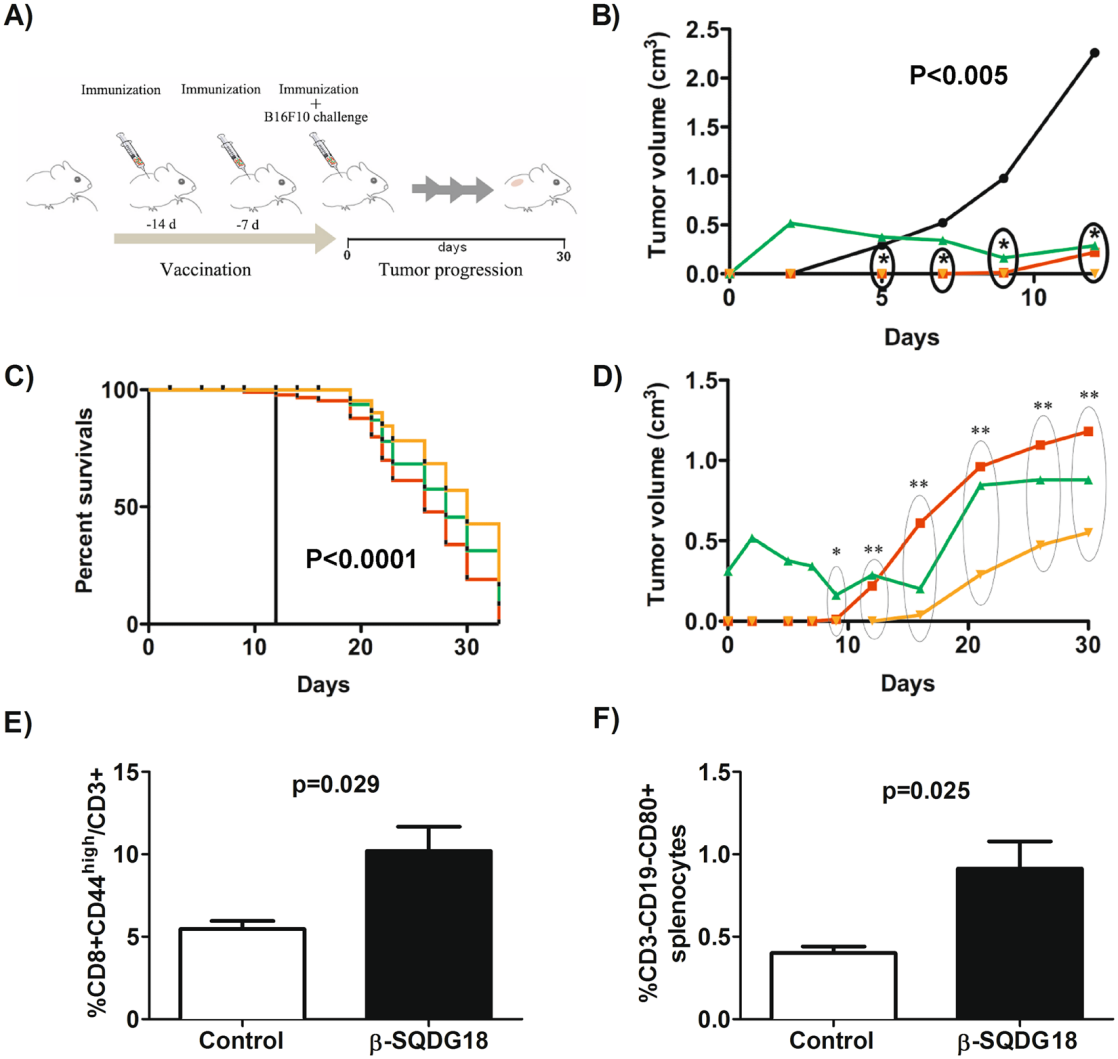
Protective effect of hgp100_25–33_ peptide formulated with **s**ynthetic β-SQDG18 as adjuvant in an experimental model of anti-melanoma vaccine. (**A**) Experimental design of immunization in C57BL/6 J mice challenged by 10^5^ B16F10 melanoma cells (day 0). The animals were pretreated with the adjuvanted antigen hgp100_25–33_ peptide (100 μg mouse/injection) on days −14, −7 and 0. For the experiment with β-SQDG18 (600 μg mouse/injection), the antigen was vigorously mixed with a homogeneous suspension of the molecule prior to injection. Mice immunized with the melanoma epitope in association to either CPG oligodeoxynucleotide (30 μg/injection) or Freund’s adjuvants (1:1 vol/vol) were used as positive controls. Unimmunized mice were used as negative control. Tumour volume of melanoma lesions and survival rate were monitored for 30 days after challenging. Each group was composed of eight animals; (**B**) Tumour growth and (**C**) percent survival in the four groups of mice. Statistically significant difference between the negative control and each of the three immunized groups of mice is indicated. Black line: negative control; Red line: antigen plus β-SQDG18; Green line: antigen plus Freund’s adjuvant; Orange line: antigen plus CpG; (**D**) Detail of tumour growth in response to vaccination with the three adjuvants. Groups are indicated by colours as above Asterisks indicate statistically significant differences in comparison to negative control. *P < 0.01; **P < 0.002; (**E**) Percentage of CD8^+^CD44^high^ memory T cell in splenocytes of tumour-challenged mice in the negative control group or after vaccination by β-SQDG18 as adjuvant (black column); (**F**) Percentage of CD3-CD19-CD80^+^ cells in splenocytes of tumour challenged mice in the negative control group and after vaccination by β-SQDG18 as adjuvant (black column).

In a second strategy of immunization, we tested β-SQDG18 (**6**) in an experimental model of cancer vaccine against a murine B16F10 melanoma cell line, that is widely used for therapeutic evaluation^39^. Activating immune responses against cancer is considered a promising therapeutic approach even if the clinical application of therapeutic cancer vaccines is currently hampered by the difficulties in the selection of effective immunization protocols. In fact, the weak antigenicity of many tumor antigens requires the association with robust adjuvants to boost the cell-mediated immune response^40^. In our experiment, β-SQDG18 (**6**) was tested in mice challenged with the human gp100_25–33_ (hgp100_25–33_) peptide, an antigen derived from a melanocyte differentiation protein^41, 42^. The adjuvant effect of β-SQDG18 (**6**) was compared with those of Freund’s and CpG Oligodeoxynucleotide (CpG) adjuvants. Vaccines formulated with CpG have shown to elicit both cellular and humoral responses so that the oligonucleotide has been tested in clinical and preclinical trials^43, 44^. In agreement with a previous protocol of vaccination showing a protective effect of hgp100_25–33_ peptide in association with CpG^45^, we immunized mice with the melanoma epitope for two weeks before challenging with B16F10 melanoma cells (1 × 10^5^ cells *per* mouse) (Fig. 4). In line with the previous results^45^, the three treatments reduced tumour growth and prolonged survival in comparison to control animals challenged only with B16F10 cells. Association of β-SQDG18 to hgp100_25–33_ peptide induced *in vivo* expansion of both memory lymphocytes and APCs, as indicated by the increase in the percentage of CD8^+^CD44 high T lymphocytes and CD3-CD19-CD80^+^ cells among splenocytes in immunized mice in comparison with unvaccinated tumour-challenged animals (Fig. 4E and F). The protective activity of vaccination with hgp100_25–33_ in association with β-SQDG18 (**6**) was comparable with the effect of the other two immunizing protocols (Fig. 4), thus providing the final proof of the efficacy of this molecule in the vaccine formulation.

## Discussion

For more than 70 years, aluminum salts have been the only adjuvants licensed for human use worldwide^46^. In the last decades, the improved understanding of how innate mechanisms influence the adaptive immunity has made possible the development of new adjuvants in a more rational design. A major breakthrough has been the approach of stimulating antigen-presenting cells, mostly DCs, that efficiently engage the adaptive immune system^1–5^. Screening of a library of extracts from marine microalgae for stimulation of human monocyte-dendritic cells (hMo-DCs) gave positive hits associated with α-sulfoquinovosides (Fig. 1), natural glycolipids wide spread in photosynthetic organisms but very rich in microalgae. In the last years, the emerging role of small molecules and lipids in immunogenicity has gained significant research traction^1, 47–55^. Marine lipids often do not have counterpart in terrestrial organisms and are considered a potential source of novel immunogenic compounds. The algal products showed only a little activity but modification of their structure led to the synthetic analog β-SQDG18 (**6**) that induces maturation of human DCs, production of inflammatory cytokines (Fig. 2) and boosts the immune protective response *in vivo* (Figs 3 and 4). Considering the difference in the stimulatory activities between the synthetic molecule and the natural products, introduction of saturated fatty acids bound to glycerol and β-configuration of the anomeric carbon are the major determinants of the DC response. This is likely due to the resemblance of the synthetic molecule with bacterial lipids that, differently from those found in mammalians and plants, are mainly made of saturated alkyl chains.

The DC activation by β-SQDG18 (**6**) is TLR-independent as the molecule did not activate TLR2- or TLR4-dependent cell lines (Fig. 2D). In consideration of the chemical structure of the sulfolipid and the close analogies with LPS-related adjuvants (e.g. MPLA)^56^, this result was rather surprising. However, DCs maturation does not necessarily require TLR-mediated triggering and a few bacterial molecules, also currently considered as vaccine adjuvants, can directly stimulate DC function without activation of TLR signaling pathways^57^. As the cell-based assay measures LPS response at the ng range, the absence of appreciable signal also excludes the contamination of the tested samples of β-SQDG18 (**6**) with small amounts of the lipopeptides.

DC stimulation is of central interest in the development of preventive and therapeutic vaccines against cancer^19, 40^. As shown in Fig. 4, β-SQDG18 (**6**) reduced tumor size and prolonged animal survival in an experimental model of vaccination against melanoma in mouse. Use of the sulfolipid gave results very similar to those achieved by well-established adjuvants, such as CpG and Complete Freund’s Adjuvant. Notably, vaccination with β-SQDG18 and CpG, but not Freund’s, also resulted in the delay of tumour growth in the first ten days after challenging (Fig. 4). Stimulation of DCs is extremely well suited to activate T-cell response in patients with cancer and, very recently, the clinical efficacy of this strategy has been proven in an innovative approach using nanoparticles of RNA–lipid complexes^58^. The demonstration that β-SQDG18, as CpG, targets DCs and induces their activation and maturation suggests that the protective effect of both adjuvants in the first days after vaccination correlates with the enhancement of the immunogenicity of hgp100_25–33_. These results are very encouraging for further testing of β-SQDG18 in anticancer formulations.

Taken together, these data pave the way to a new class of adjuvants based on a single molecule. Formulation of these products offers a few technical advantages because it does not require additional products (i.e. surfactants) and the dosage of the single product is straightforward. This simplifies the evaluation of the biological response too. Importantly, we also demonstrated that preparation of the sulfolipid molecule can be scaled by standard chemical methods in order to fulfil the supply need for clinical development. Chemical modifications of the product and optimization of the formulation protocol will probably allow reduction of the amount of β-SQDG18, thus further improving safety and efficiency of vaccination protocols.

## Methods

All methods were performed in accordance with the local regulations and relevant guidelines of CNR –Institute of Biomolecular Chemistry and Department of Internal and Experimental Clinic of the University of Campania. Mice were handled and treated according to the guidelines provided in Ministero della Salute D.lgs 26/2014 under the supervision of the Animal Facility OPBA of the National Health Service - University Hospital San Martino of Genoa.

### General Experimental Procedures

1D- and 2D-NMR spectra were recorded on AVANCE*™*III HD 400 MHz spectrometer (Bruker Bio-Spin, Fällanden, Swiss) and Bruker DRX 600 MHz spectrometer (Bruker Bio-Spin, Fällanden, Swiss) equipped with a TXI CryoProbe in CDCl_3_ and CD_3_OD (δ values are referred to CHCl_3_ and CH_3_OH at 7.26 and 3.34 ppm respectively). Mass analyses were aquired on a Micromass® Q-Tof micro™ mass spectrometer coupled with an Alliance HPLC System (Waters Corp., Milford, MA, USA) or on a Q Exactive™ Hybrid Quadrupole-Orbitrap™ Mass Spectrometer (Thermo Scientific,Waltham, MA, USA) coupled with an 1290 Infinity UPLC System (Agilent Technologies, Santa Clara, CA, USA). Solvents (LC-MS grade), TLC plates (Kieselgel 60 F_254_) and silica gel powder (Kieselgel 60, 0.063–0.200 mm) were from Merck (Darmstaadt, Germany). Other solvents were purchased from VWR (Radnor, PA, USA). All the reagents were obtained from Sigma-Aldrich (Saint-Louis, Mo, USA) and used without any further purification.

### Algal culturing

*Thalassiosira weissflogii* was cultured in 70 L photobioreactors filled with 0.22 μm FSW enriched with f/2 medium. Cultures were gently bubbled with filtered ambient air and grown in a climate chamber at 20 °C under 12 h:12 h light:dark cycle (100 μmol photons·m^2^·s^−1^). Cells were harvested at the stationary phase by centrifugation at 3750 rpm for 10 min at 4 °C in a swing-out rotor.

### Extraction and isolation of complex lipids from marine diatoms

For preliminary biological screening, microalgal extracts were prepared and fractionated according our previous protocol^59^. Briefly methanol extracts were obtained from wet pellet (about 200 mg) and fractionated (about 20 mg) on HRX column by using five eluents, i.e. A, H_2_O 100%; B, MeOH/ H_2_O 1:1, C, ACN/ H_2_O 7:3; D, ACN 100% and E, CH_2_Cl_2_/MeOH 9:1. α-SQDG eluted in fraction C. For preparative purposes, MeOH extract of *T. weissflogii* was chromatographed on Sephadex LH-20 column by using CHCl_3_/MeOH (1:1) as eluent. Fraction containing sulfoquinovosides was further purified by radial silica chromatography on Chromatotron (T-Squared Technology Inc, San Bruno, CA, USA) by CHCl_3_:MeOH and CHCl_3_:MeOH:H_2_O, 65:25:4. Pure α-SQDGs were characterized by NMR and MS methods.

### LC-MS/MS Analysis of natural α-SQDG

Species composition was identified by LC-MS/MS according to our recent report^26^. Briefly, UPLC separation was performed on a Kinetex Biphenyl (2.6 µm, 150 × 2.1mm) column (Phenomenex, Torrance, CA, USA) by using a binary mobile phase of water and methanol (MeOH). The elution program (flow rate 0.3 mL/min) was from 40% to 80% MeOH in 2 min, then 100% MeOH in 13 min and 100% MeOH for other 7 min. Full scan mass spectra (ESI in negative ion mode) were acquired in the *m/z* range 150–2000 with a resolution of 70000. MS/MS experiments on monoisotopically isolated precursor ions were performed with a normalized collision energy between 20 and 40.

### Glycolipid synthesis

Synthetic analogs **3**-**5** were prepared according to Manzo *et al*.^27^. Synthesis of compounds **6** is described in the Supplementary Information. 1,2-distearoyl-3-O-β-D-sulfoquinovosy]-(R/S)-glycerol (**6**): 1,2-distearoyl-3-*O*-[(2′,3′,4′-tri-acetyl-)-β-D-sulfoquinovosyl]-(*R*/S)-glycerol (0.40 g, 0.00040 mol) was dissolved in aq. ethanol (85%) (30 mL), hydrazine monohydrate (0.16 g, 0.00336 mol) was added, and the reaction mixture was stirred for 3 h at 44 °C. After evaporation under a stream of nitrogen, the mixture was purified by silica gel chromatography using a gradient of chloroform/methanol to give 1,2-distearoyl-3-*O*-β-D-sulfoquinovosy]-(*R*/S)-glycerol (**6**) (0.26 g, 0.00030 mol, 70%) as a white solid, m.p. 88–92 °C; R_*f*_ (chloroform/methanol 7:3) = 0.15; IR (liquid film) v_max_ 3400, 2940, 2862, 1750, 1351, 1343 cm;^**-**1 1^H-NMR (400 MHz, CDCl_3_/CD_3_OD 1/1): ^1^H-NMR (400 MHz, CDCl_3_/CD_3_OD 1/1): δ 5.29 (1H, m, m, H-2), 4.47 (1 H, m, H-1a), 4.34 and 4.32 (each for 1 H, d, 7.8 Hz, H-1′), 4.19 (1 H, m, H-1b), 4.13–4.03 (1 H, m, H-1a), 3.79–3.75 (2 H, m, H-3b, H-5′), 3.42 (1 H, m, H-3′), 3.32 (1 H, m, H-6′a), 3.26 (1 H, m, H-2′), 3.14 (1 H, m, H-4′), 2.98 (1 H, m, H-6′b), 2.43–2.35 (4 H, m, α-methylene), 1.69–1.58 (4 H, m, β-methylene), 1.43–1.29 (acyl chain), 0.94 (6 H, overlapped, 2 CH_3_); HRESIMS *m/z* 849.5757 [M-K]^−^ (calcd for C_45_H_85_O_12_S^−^, 849.5767).

### Preparation of Dendritic Cells

For each assay human peripheral blood mononuclear cells were isolated from two healthy donors by routine Ficoll density gradient centrifugation. Monocytes were purified from human peripheral blood mononuclear cells using MACS CD14 microbeads (Miltenyi Biotech, Auburn, CA, USA) according to the manufacturer’s recommendation. Purity was checked by staining with a FITC-conjugated anti-CD14 antibody (Milteny Biotech, Auburn, CA, USA) and FACS analysis and was routinely found to be greater than 98%. Immature DCs were obtained by incubating monocytes at 1∙10^6^/mL in RPMI 1640 medium supplemented with 10% fetal calf serum, 1% L-glutamine 2 mM, 1% penicillin and streptomycin, human IL-4 (5 ng/mL) and human GM-CSF (100 ng/mL) for five days.

### **C**ells Staining and stimulation

After five days in culture, surface staining was performed on monocyte-derived dendritic cells (MoDC) for flow cytometry analysis. In the initial screening assay moDCs were stained to check the maturation state with mouse anti-human HLA-DR-PE (BD PharMingen, Haryana, India), CD86-FITC, CD3-PerCP (Milteny Biotech, Auburn, CA, USA) and CD14-FITC monoclonal antibodies. Then the experiments were conducted by using the following conjugated mAbs from BD Biosciences (Milano, Italy): CD14Fitc, CD1a-BUV121, CD86 BV650, CD83BUV737, HLA-DRBV786, CD11c-BUV395, CD3 BV510, CD54Pe, CD1c Alexa Fluor 647, CD4- PeCys7, and analyzed by flow-cytometer on LSRFortessa*™* X*-*20 cell analyzer (BD Bioscience, Frankin Lake, NJ, USA) according to standard protocol. MoDC were then incubated with natural and synthetic compounds in 12-wells. Stimulation with Pam2CSK4 1 µg/mL (Invivogen, San Diego, CA, USA) was used as positive control. Cells treated only with vehicle (DMSO) were used as control. Release of cytokines was measured by LUMINEX^®^ platform by Human IL-12p70 Luminex Performance Assay and Human TNF-alpha Luminex Performance Assay according to manufacturer’s instructions (R&D Systems, Minneapolis, MN, USA). After 24 hours, expression of all surface markers was estimated again by fluorochrome-conjugated antibodies.

### Real Time PCR analysis

Total RNA was isolated using Trizol Reagent, according to the manufacturer’s protocol. RNA quantity and purity were measured with a NanoDrop 2000 spectrophotometer (Thermo Scientific,Waltham, MA, USA). Sample purity was checked by A260/A280 ratios between 1.80 and 2.00. Extracted RNAs from all preparations were in this range. Cytokines mRNA expression was measured by quantitative Real Time-PCR. Results are expressed as means ± SD. All data were analyzed by one way ANOVA followed by the Tukey test for multiple comparison test. A p-value less than 0.05 was considered as statistically significant. All analyses were performed using the GraphPad Prism 4.00 for Windows software (GraphPad Software, San Diego California, USA).

### PCR Array analysis

About 200 ng RNA was subjected to reverse transcription reaction using the RT2 first strand kit (Qiagen, Hilden, Germany) according to the manufacturer’s instructions. The qRT-PCR analysis was performed in triplicate using the RT2 Profiler PCR Array kit (Qiagen, Hilden, Germany), in order to analyse the expression of inflammatory cytokines and receptors on dendritic cells stimulated. Plates were run on an Applied Biosystem ViiA 7 Real-Time PCR System (384 well blocks) (Thermo Scientific,Waltham, MA, USA) by Standard Fast PCR Cycling protocol with 10 µl reaction volumes. Cycling conditions used were: 1 cycle initiation at 95.0 °C for 10 min followed by amplification for 40 cycles at 95.0 °C for 15 s and 60.0 °C for 1 min. Amplification data were collected via A ViiA 7 RUO Software (Applied Biosystems). The cycle threshold (Ct)-values were analyzed with PCR array data analysis online software (Qiagen, Hilden, Germany).

### TLR assay

Human TLR-4/NF-kB/SEAP and TLR2/ NF-kB/SEAP (SEAPorter™) HEK 293 reporter cells (Novus Biologicals, Littleton, CO, USA) were plated in 24-well plates at 2×10^5^ cells/well. After 16 hours, for functional assays, TLR4 cells, cells were transiently transfected with MD2/CD14 plasmid vector (Invivogen, San Diego, CA, USA) for 24 hours. Cells were then stimulated with β-SQDG18 for 24 hours. LPS for TLR-4 cell line and Pam2CSK4 for TLR-2 cell line were used as positive control under the same conditions. SEAP was analyzed using the SEAPorter Assay (Novus Biologicals, Littleton, CO, USA) in according to manufacturer’s instructions. Quantitative data (ng/mL) were obtained by a standard curve for the SEAP protein.

### Immunization protocol

Immunization of C56BL/6 mice (four groups of three individuals) was carried out by injecting twice 5 µg Ovalbumin (OVA) (Hyglos GmbH, Bernried am Starnberger See, Germany) together with 2.5 mg β-sulfoquinovoside mixture (**6**) according to published protocols^60^. OVA-specific Ig production was measured by ELISA according to Leung S *et al*.^61^. Titermax and Complete Freund’s Adjuvant (Sigma Aldrich, St. Louis, MO, USA) were used according to manufacturer instructions. Statistical analyses were performed by ANOVA using the GraphPad Prism 4.0 Software (GraphPad Software, Inc, La Jolla, CA).

### Comparative analysis of cancer vaccine formulations

Experiments were carried out according to the published protocol^45^ by challenging C57BL/6 J mice with subcutaneously injection of B16F10 melanoma cells (1 × 10^5^ cells/mouse). Prior to challenge, the animals were immunized three times at days −14, −7 and 0 by subcutaneous injection of hgp100_25–33_ peptide (100 μg/mouse injection) in association with one of the following products: CpG (30 μg) (Tib MolBiol, Genoa, Italy), Freund’s adjuvant (1:1 vol/vol) (Sigma Aldrich, St. Louis, MO), β-SQDG18 (600 μg). Formulations were carried out according to manufacturers’ instructions. For β-SQDG18, the melanoma antigen was simply added to a stable emulsion of the sulfolipid in phosphate buffer prior to injection. Experiments were carried out on groups of 8 animals each. Tumor masses were measured with a caliper at 2–3 days intervals by measuring long and short axes. Volume was calculated according to the formula: tumor volume = ½ (length × width^2^) in cm^3^. Mice were sacrificed when either tumors reached > 1 cm^3^, when ulceration/bleeding developed, or after 30 days from the tumor challenge. Mice treated with hgp100_25–33_ peptide alone prior to challenging with B16F10 cells (1 × 10^5^ cells/mouse) were used as negative control. Statistical analyses were performed by the Mann-Whitney unpaired T test for nonparametric measures using the GraphPad Prism 4.0 Software (GraphPad Software, Inc, La Jolla, CA).

### Analysis of splenocytes from B16F10 challenged mice

Spleens were removed from sacrificed mice according to Kalli *et al*.^45^. Frequency of memory T lymphocytes was assessed *ex vivo* on splenocytes stained by the following conjugated mAbs: CD3-BV510 (BD PharMingen, Haryana, India), CD4-APC-eFluor 780, CD8-PE-Cy7, CD44- PE (Thermo Scientific,Waltham, MA, USA). To assess *ex vivo* the percentages of non-lymphoid APCs (monocytes and DCs), splenocytes were labelled with the following conjugated mAbs: CD80- FITC (Biolegend, San Diego, CA, USA), HLA-DR-PE, CD86-APC (Thermo Scientific,Waltham, MA, USA). The samples were analyzed by a FACS Canto II flow-cytometer (BD Bioscience, Frankin Lake, NJ, USA) using FACS DIVA software. In both analyses, splenocytes were stained with Live/dead fixable violet dead cell stain (Thermo Scientific,Waltham, MA, USA) to exclude dead cells. Statistical analyses were performed by the Mann-Whitney unpaired T test for nonparametric measures using the GraphPad Prism 4.0 Software (GraphPad Software, Inc, La Jolla, CA).

## Electronic supplementary material

Supplementary Information
