# Supplementary material for: A new marine-derived sulfoglycolipid triggers dendritic cell activation and immune adjuvant response
Source: Sci Rep. 2017 Jul 24;7:6286. doi: 10.1038/s41598-017-05969-8 (PMC5524952; doi:10.1038/s41598-017-05969-8)
Supplement: Supplementary file 1 — Supplementary Information [file 41598_2017_5969_MOESM1_ESM.doc]

**SUPPLEMENTARY INFORMATION**

**A new marine-derived sulfoglycolipid triggers dendritic cell activation and immune adjuvant response**

**Emiliano Manzo^1^, Adele Cutignano^1^, Dario Pagano^1^, Carmela Gallo^1^, Giusi Barra^2^, Genoveffa Nuzzo^1^, Clementina Sansone^3^, Adrianna Ianora^3^, Konrad Urbanek^4^, Daniela Fenoglio^5,6^, Francesca Ferrera^5,6^, Cinzia Bernardi^5^, Alessia Parodi^5^, Giuseppe Pasquale^2^, Antonio Leonardi^7^, Gilberto Filaci^5,6^, Raffaele De Palma^2,8^* and Angelo Fontana^1^***

**Supplementary Figure 1**. Interleukin production by MoDCs after stimulation with α-SQDG (**2**) of *Thalassiosira weissflogii.*


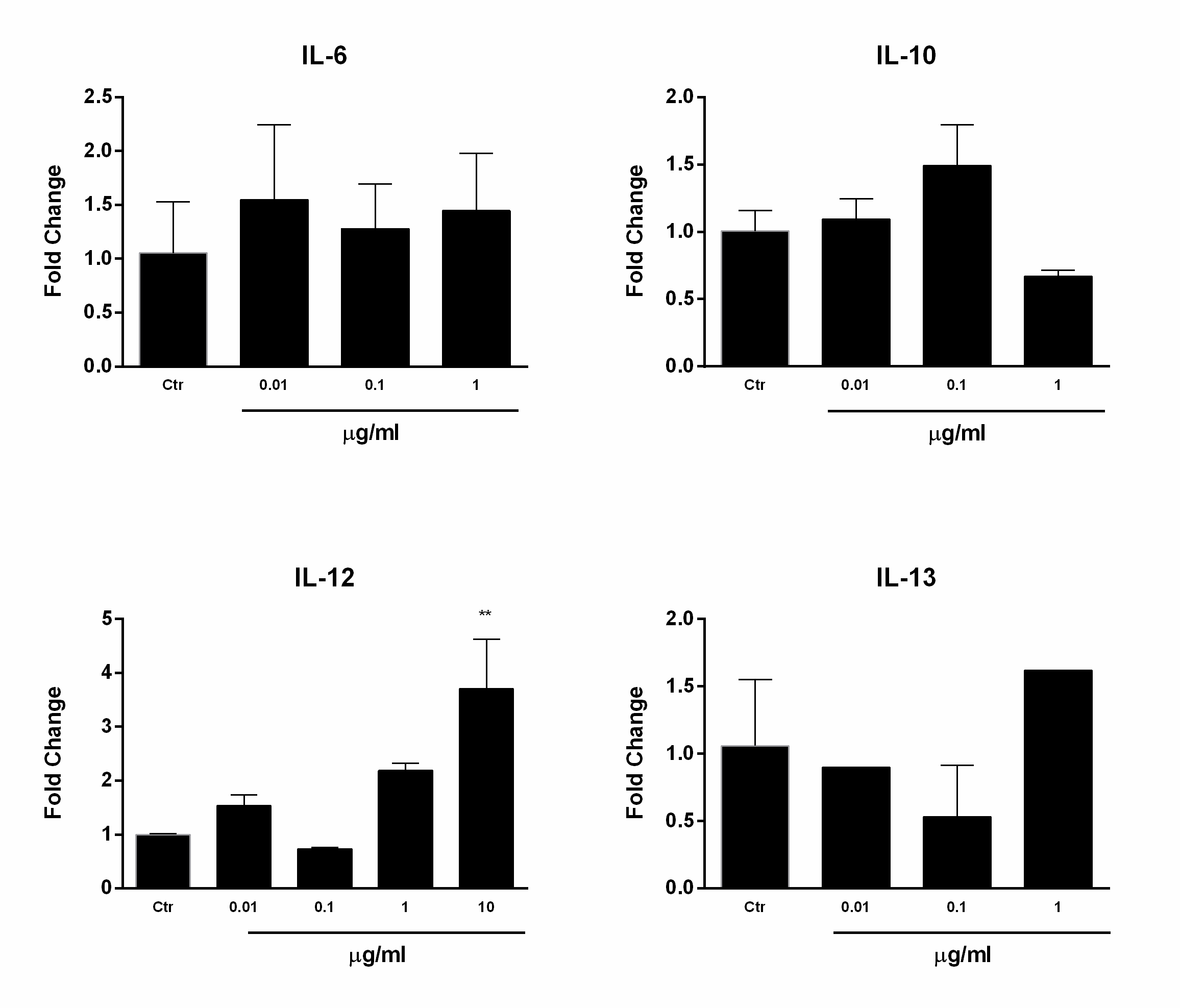


**Supplementary Figure 2**. CD86 surface expression of MoDCs after stimulation with natural (2) and synthetic (5) α-SQDG at 10 ng/mL and 100 µg/mL.


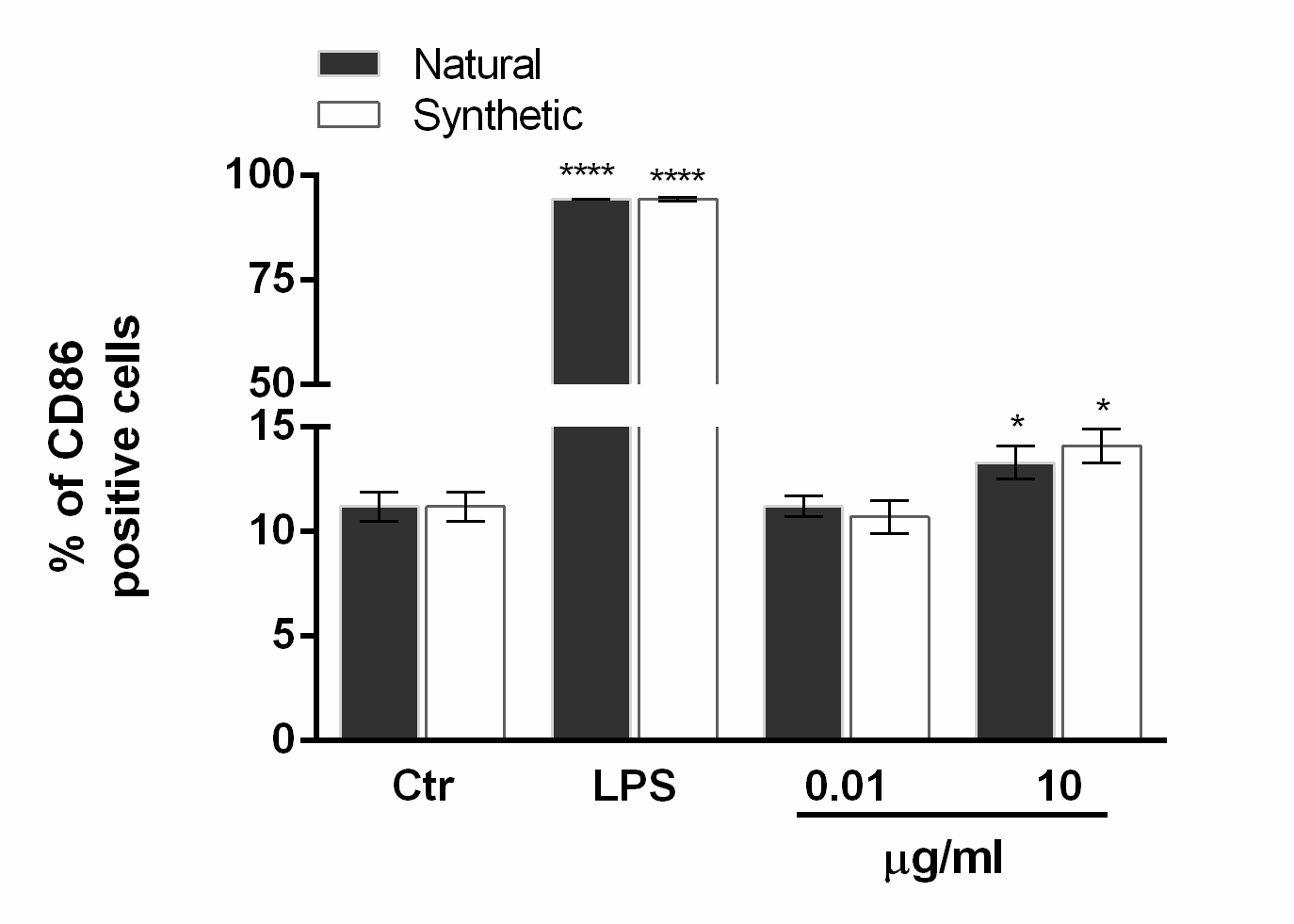


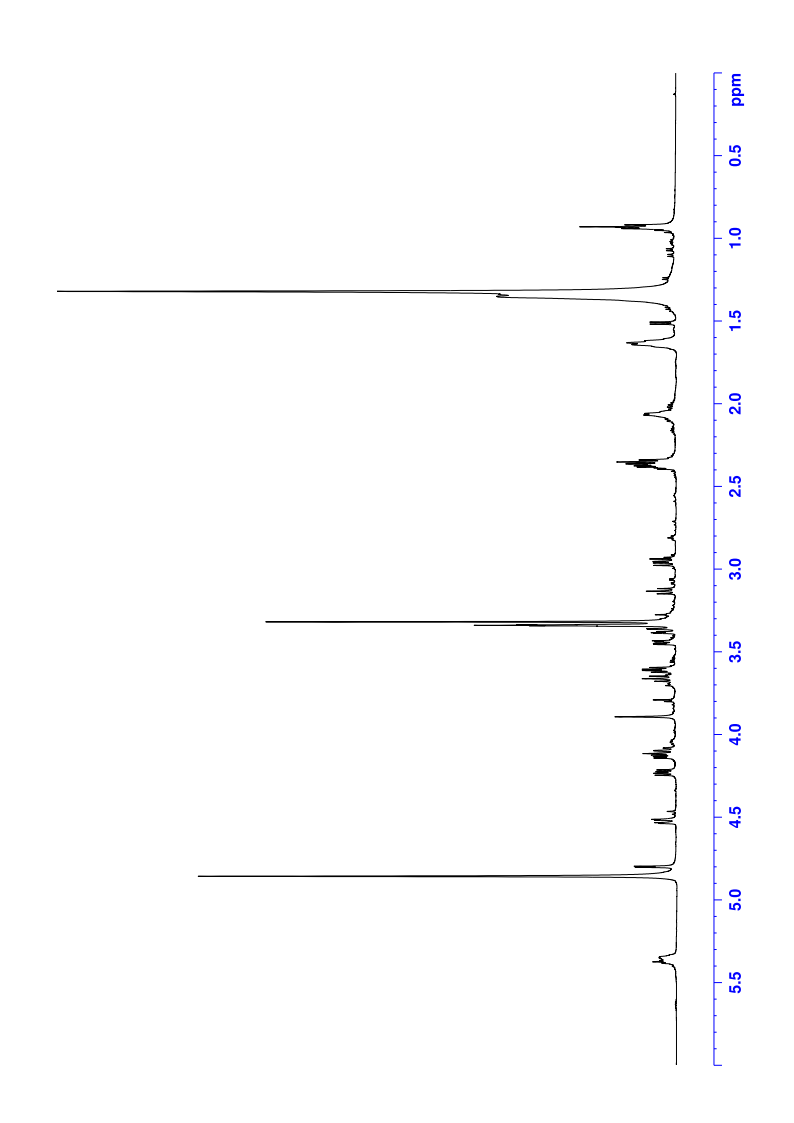
**Supplementary Figure 3**. ^1^H-NMR spectrum (600MHz, CD_3_OD) of α-SQDG from *Thalassiosira weissflogii* (**2**).

**Supplementary Figure 4**. ^13^C-NMR spectrum (600MHz, CD_3_OD) of α-SQDG (**2**) from *Thalassiosira weissflogii*.

***
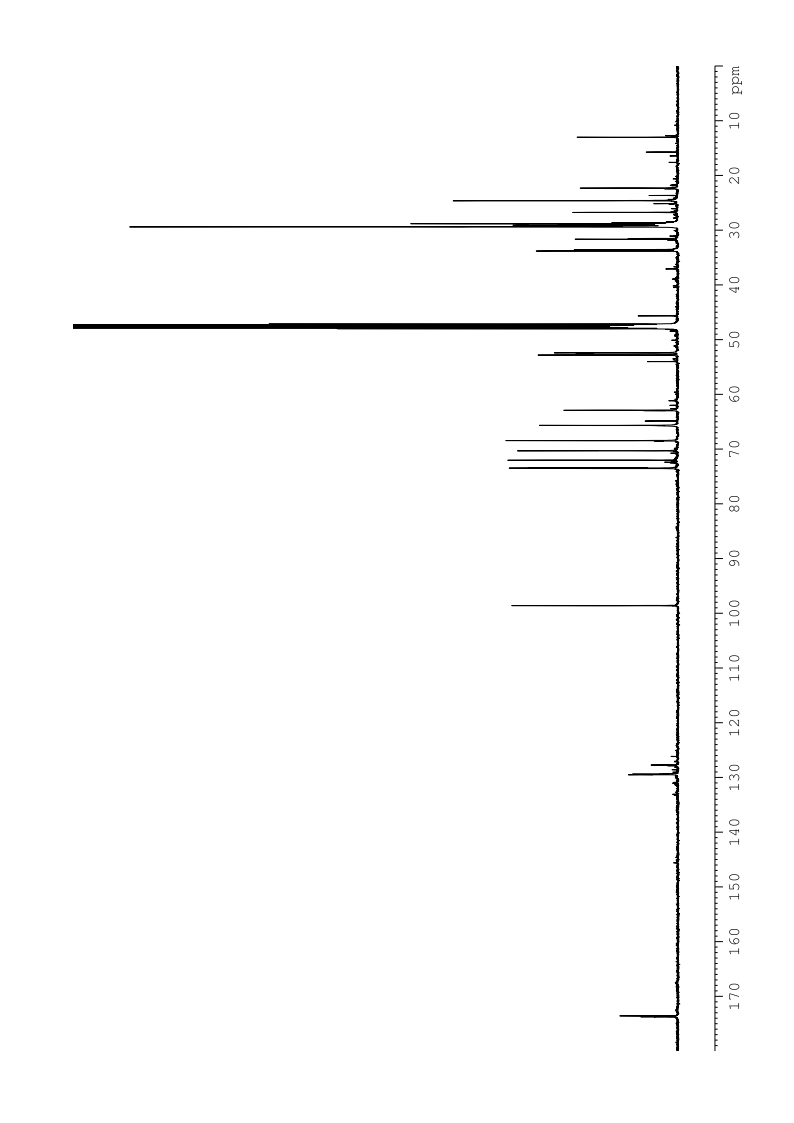
***

**Supplementary Figure 5**. Interleukin production by MoDCs after stimulation with β-SQDG18 (**6**).


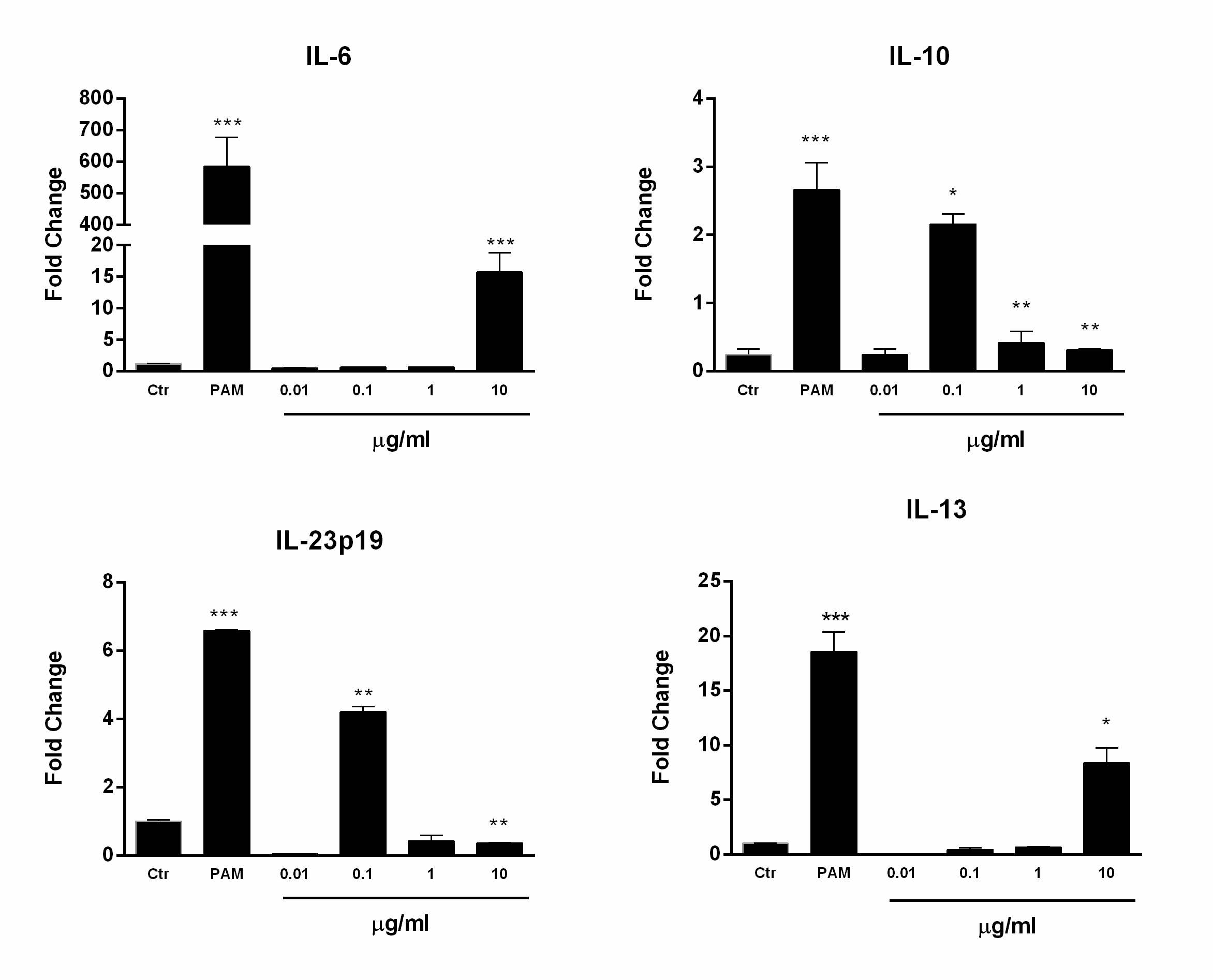


**Supplementary Figure 6.** Synthesis of 1,2-distearoyl-3-*O*-(β-sulfoquinovosyl)-*(S/R)*-glycerol (**6**).

**Supplementary Figure 7**. Complete phenothypic characterization of MoDCs by flow cytometry after stimulation with β-SQDG18 (**6**).


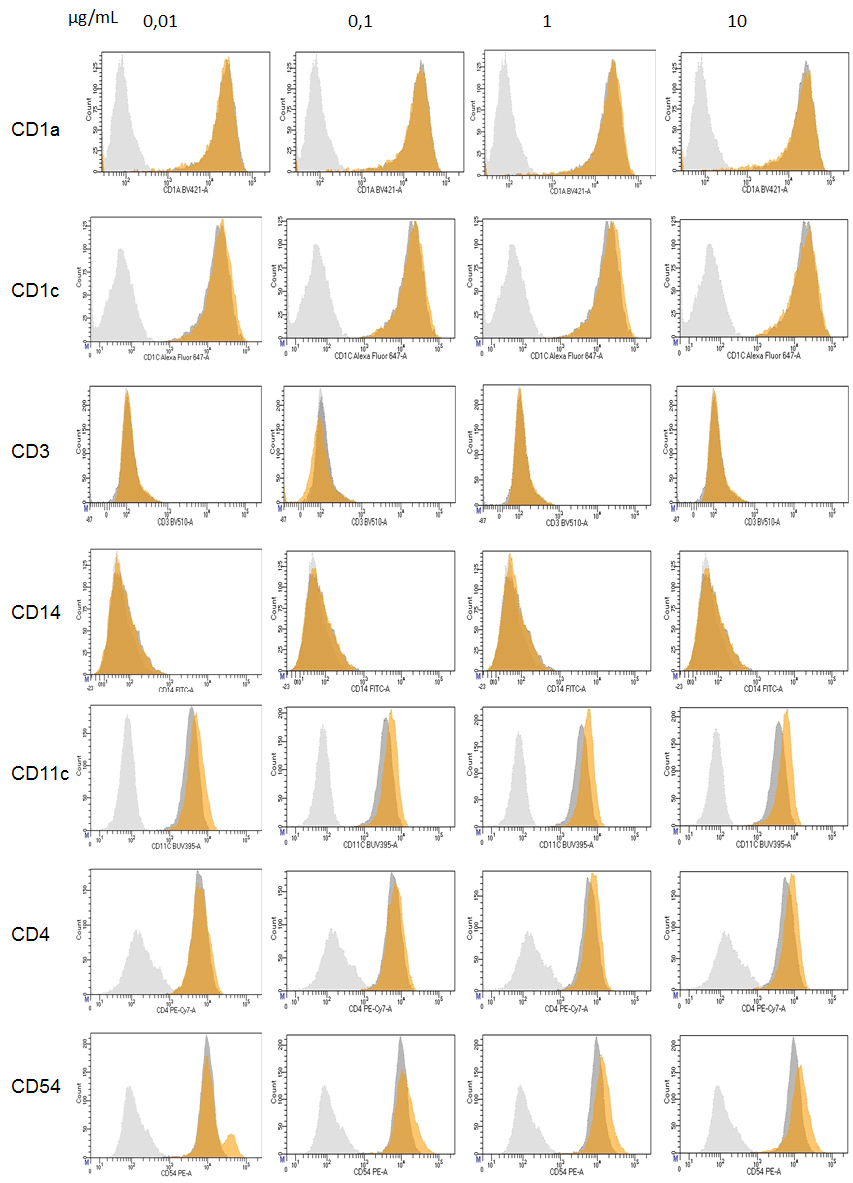


**Supplementary Figure 8:** Effect of the β-SQDG18 (**6**) on the expression of inflammatory cytokines and receptors on dendritic cells stimulated. Three independent assays were performed in triplicate and the data are expressed as mean ±S.D. Expression values greater or lower than a two-fold difference with respect to the controls were considered significant.


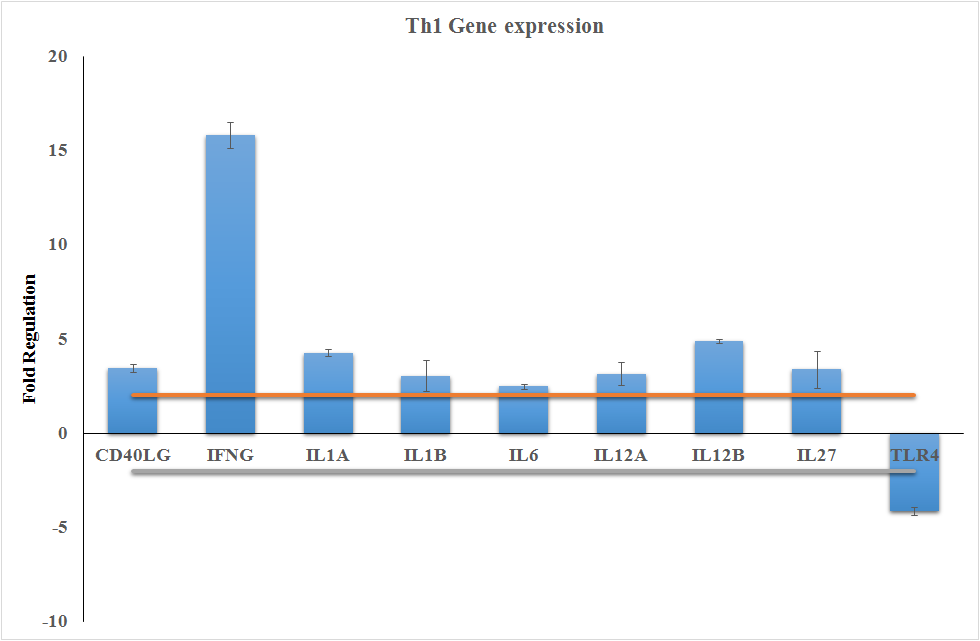


**Supplementary Figure 9:** Release of IL-12 and TNFα from DC stimulated by β-SQDG18 (10 µg/mL). Protein levels were measured by LUMINEX® platform by Human IL-12p70 Luminex Performance Assay and Human TNF-alpha Luminex Performance Assay according to manufacturer's instructions (R&D Systems)

**
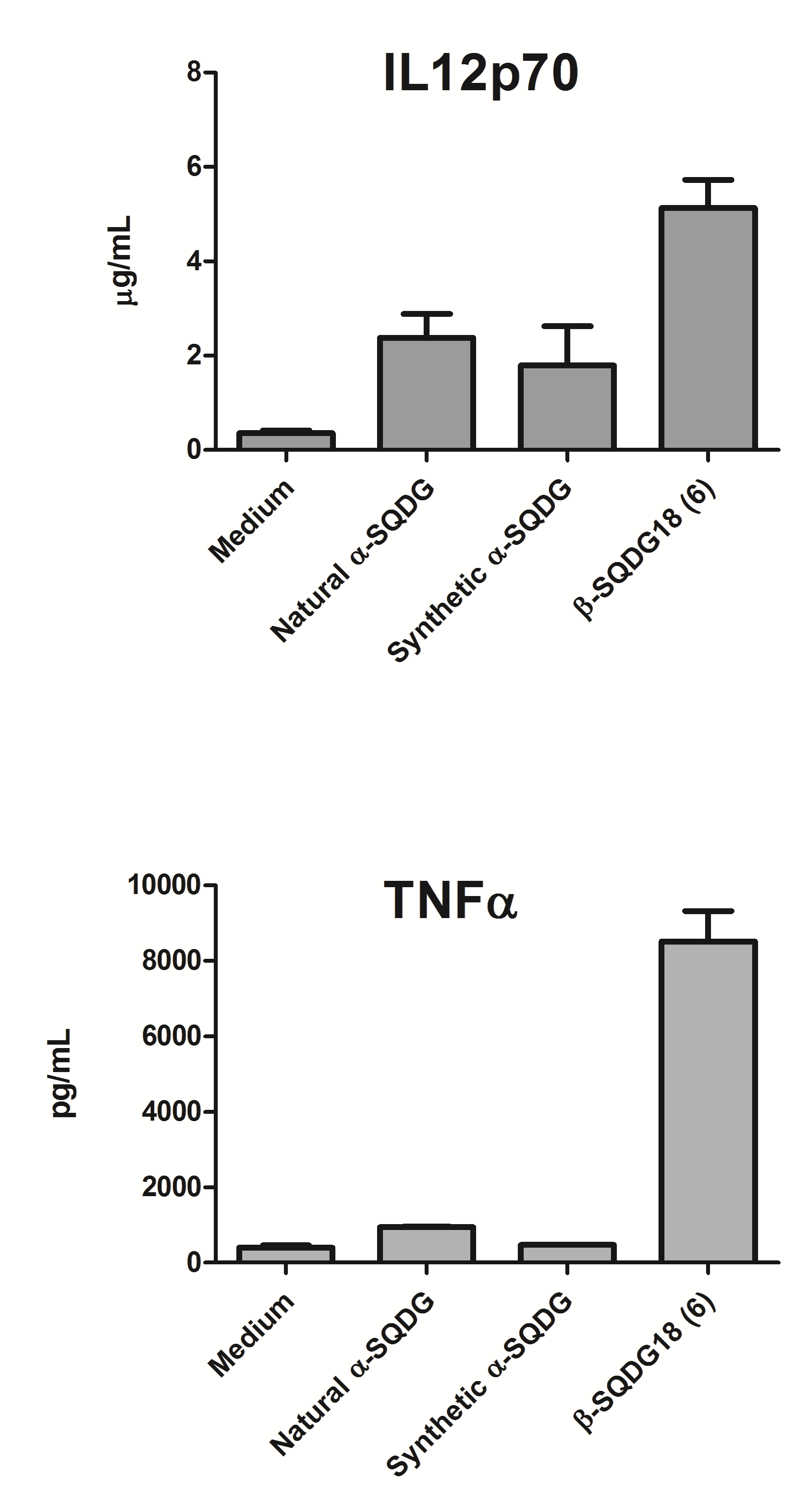
**

**Supplementary** **Table 1:** List of the 69 genes analysed by PCR array technique

| **Unigene** | **Symbol** | **Description** | **Fold Regulation** | **St. Dev.** |
| --- | --- | --- | --- | --- |
| Hs.441047 | ADM | Adrenomedullin | 1.1168 | 0.60522 |
| Hs.76152 | AQP1 | Aquaporin 1 (Colton blood group) | 1.5788 | 0.232 |
| Hs.130730 | AQP2 | Aquaporin 2 (collecting duct) | 1.8945 | 0.53796 |
| Hs.315369 | AQP4 | Aquaporin 4 | 38.9513 | 0.5705 |
| Hs.632446 | ARNT | Aryl hydrocarbon receptor nuclear translocator | 0.9594 | 0.2 |
| Hs.496487 | CA9 | Carbonic anhydrase IX | 0.9117 | 0.40697 |
| Hs.492740 | CASP1 | Caspase 1, apoptosis-related cysteine peptidase (interleukin 1, beta, convertase) | 1.5078 | 0.184554 |
| Hs.42853 | CCR2 | Chemokine (C-C motif) ligand 2 | 3.6373 | 0.18307 |
| Hs.264482 | CD40LG | CD40 ligand | 3.4614 | 0.1968 |
| Hs.486063 | CDKN1A | Cyclin-dependent kinase inhibitor 1A (p21, Cip1) | 2.2271 | 0.6656 |
| Hs.740389 | CFTR | Cystic fibrosis transmembrane conductance regulator (ATP-binding cassette sub-family C, member 7) | 4.3145 | 0.3963 |
| Hs.367437 | CHEK1 | CHK1 checkpoint homolog (S. pombe) | 1.1074 | 0.148684 |
| Hs.271791 | CRP | C-reactive protein, pentraxin-related | 7.1792 | 0.3789 |
| Hs.467020 | EDN1 | Endothelin 1 | 9.0944 | 0.236489 |
| Hs.716464 | EPO | Erythropoietin | 2.8271 | 0.3599 |
| Hs.517145 | FAS | Fas (TNF receptor superfamily, member 6) | 1.1484 | 0.77353 |
| Hs.131226 | FTH1 | Ferritin, heavy polypeptide 1 | 1.0565 | 0.53234 |
| Hs.63287 | HSP90AA1 | Heat shock protein 90kDa alpha (cytosolic), class A member 1 | 0.914 | 0.2207 |
| Hs.515162 | HSPA4L | Heat shock 70kDa protein 4-like | 1.7916 | 0.21235 |
| Hs.2490 | HSPA5 | Heat shock 70kDa protein 5 (glucose-regulated protein, 78kDa) | 1.2268 | 0.11012 |
| Hs.303649 | IFNg | Interferon, gamma | 15.816 | 0.68763 |
| Hs.592244 | IL1A | Interleukin 1, alpha | 4.2697 | 0.18 |
| Hs.370771 | IL1B | Interleukin 1, beta | 3.0549 | 0.8 |
| Hs.489786 | IL6 | Interleukin 6 (interferon, beta 2) | 2.4801 | 0.11876 |
| Hs.24529 | CXCL8 | Interleukin 8 | 1.6276 | 0.41016 |
| Hs.291363 | MMP9 | Matrix metallopeptidase 9 (gelatinase B, 92kDa gelatinase, 92kDa type IV collagenase) | 1.1361 | 0.231 |
| Hs.709456 | NBN | Nibrin | 1.33 | 0.6712 |
| Hs.700338 | NFAT5 | Nuclear factor of activated T-cells 5, tonicity-responsive | 1.0074 | 0.1156 |
| Hs.505777 | CD155 | Poliovirus receptor | 1.111 | 0.42212 |
| Hs.59214 | SQSTM1 | Sequestosome 1 | 1.6523 | 0.6134 |
| Hs.511899 | TLR4 | Toll-like receptor 4 | -4.125 | 0.22191 |
| Hs.2303 | TNF | Tumor necrosis factor | 4.1514 | 0.20382 |
| Hs.667309 | TNFRSF10A | Tumor necrosis factor receptor superfamily, member 10a | 2.016 | 0.24723 |
| Hs.645560 | TNFRSF10B | Tumor necrosis factor receptor superfamily, member 10b | 2.0773 | 0.3773 |
| Hs.80409 | TP53 | Tumor protein p53 | 3.1204 | 0.11134 |
| Hs.9701 | TXN | Thioredoxin | 1.5844 | 0.15997 |
| Hs.654465 | ULK1 | Unc-51-like kinase 1 (C. elegans) | 1.3264 | 0.12779 |
| Hs.596052 | VEGFA | Vascular endothelial growth factor A | 6.1564 | 0.24282 |
| Hs.444356 | IL10 | Interleukin 10 | -4.6565 | 0.23429 |
| Hs.271510 | IL11 | Interleukin 11 | -1.2223 | 0.34408 |
| Hs.523836 | IL12A | Interleukin 12A (natural killer cell stimulatory factor 1, cytotoxic lymphocyte maturation factor 1, p35) | 3.158 | 0.6119 |
| Hs.727017 | IL12B | Interleukin 12B (natural killer cell stimulatory factor 2, cytotoxic lymphocyte maturation factor 2, p40) | 4.914 | 0.1057 |
| Hs.525600 | IL13 | Interleukin 13 | -2.5788 | 0.104213 |
| Hs.192374 | IL15 | Interleukin 15 | 1.9633 | 0.2431 |
| Hs.90093 | IL16 | Interleukin 16 | 10.7916 | 0.18751 |
| Hs.135554 | IL17A | Interleukin 17A | 7.2268 | 0.39669 |
| Hs.743241 | IL17F | Interleukin 17F | 3.1779 | 0.1233 |
| Hs.152983 | IL18 | Interleukin 18 (interferon-gamma-inducing factor) | 15.816 | 0.9749 |
| Hs.856 | IL1A | Interleukin 1, alpha | 4.2697 | 0.3169 |
| Hs.1722 | IL1B | Interleukin 1, beta | 3.0549 | 0.24083 |
| Hs.126256 | IL1RN | Interleukin 1 receptor antagonist | 2.4801 | 0.20131 |
| Hs.654458 | IL2 | Interleukin 2 | 5.6276 | 0.235 |
| Hs.624 | IL21 | Interleukin 21 | 1.5234 | 0.35 |
| Hs.2795 | IL22 | Interleukin 22 | -1.614 | 0.49338 |
| Hs.632486 | IL23A | Interleukin 23, alpha subunit p19 | 1.1361 | 0.1531 |
| Hs.297413 | IL24 | Interleukin 24 | 1.676 | 0.1 |
| Hs.192649 | IL27 | Interleukin 27 | 3.3893 | 0.9881 |
| Hs.492208 | IL3 | Interleukin 3 (colony-stimulating factor, multiple) | 2.0074 | 0.9298 |
| Hs.371987 | IL4 | Interleukin 4 | 1.0136 | 0.284411 |
| Hs.406515 | IL5 | Interleukin 5 (colony-stimulating factor, eosinophil) | -1.3693 | 0.7684 |
| Hs.177766 | IL6 | Interleukin 6 (interferon, beta 2) | 2.0873 | 0.65297 |
| Hs.180909 | IL7 | Interleukin 7 | 4.7111 | 0.46186 |
| Hs.171844 | CXCL8 | Interleukin 8 | 4.0097 | 0.3713 |
| Hs.16184 | IL9 | Interleukin 9 | 2.579 | 0.6654 |
| Hs.631709 | LIF | Leukemia inhibitory factor (cholinergic differentiation factor) | -1.0497 | 0.2005 |
| Hs.655354 | LTA | Lymphotoxin alpha (TNF superfamily, member 1) | 1.3931 | 0.11343 |
| Hs.519842 | LTB | Lymphotoxin beta (TNF superfamily, member 3) | 38.0356 | 0.5543 |
| Hs.414795 | MIF | Macrophage migration inhibitory factor (glycosylation-inhibiting factor) | 2.6527 | 0.1507 |
| Hs.473721 | MSTN | Myostatin | 5.0465 | 0.7455 |

**Preparation of compound 6**

**3-*O*-[(2’,3’,4’,6’-tetra-acetyl)-β-D-glucosyl]-(*R*/S)-glycerol**: 1,2-isopropyliden-3-*O*-[(2’,3’,4’,6’-tetra-acetyl)-β-D-glucosyl]-(*R*/S)-glycerol (5.5 g, 0.012 mol)^29^ was dissolved in acetonitrile (40 mL) and 5 equiv. of zinc nitrate hexahydrate was added; the reaction mixture was heated at 50°C and kept under stirring for 6 h; after evaporation of the organic solvent under reduced pressure, the mixture was partitioned between water and chloroform, the organic phase was evaporated and purified by silica gel chromatography using a gradient of petroleum ether/ethyl acetate to give 3-O-[(2’,3’,4’,6’-tetra-acetyl)-β-D-glucosyl]-(*R*/S)-glycerol (3.9 g, 0.009 mol, 77%) as pale yellow oil. Spectral data were identical to the literature; HRESIMS 445.1319 [M+Na]^+^ (calcd for C_17_H_26_O_12_Na, 445.1322).

**1,2-distearoyl-3-*O*-[(2’,3’,4’,6’-tetra-acetyl)-β-D-glucosyl]-(*R*/S)-glycerol**: 3-O-[(2’,3’,4’,6’-tetra-acetyl)-β-D-glucosyl]-(*R*/S)-glycerol (2.1 g, 0.005 mol) was dissolved in anhydrous dichloromethane (20 mL) prior to addition of stearic acid (2.9 g, 0.01 mol), dicyclohexylcarbodiimide (2.04 g, 0.010 mol) and DMAP (0.12 g, 0.0010mol) under argon. The reaction mixture was stirred overnight at room temperature; after evaporation under reduced pressure, the mixture was purified by silica gel chromatography using a gradient of petroleum ether/diethyl ether to give 1,2-distearoyl-3-*O*-[(2’,3’,4’,6’-tetra-acetyl)-β-D-glucosyl]-(*R*/S)-glycerol (4.28 g, 0.0045 mol, 90%) as pale yellow oil. Spectral data were identical to the literature; HRESIMS 977.6545 [M+Na]^+^ (calcd for C_53_H_94_O_14_Na, 977.6541).

**1,2-distearoyl-3-*O*-β-D-glucosyl-(*R*/S)-glycerol**: 1,2-distearoyl-3-*O*-[(2’,3’,4’,6’-tetra-acetyl)-β-D-glucosyl]-(*R*/S)-glycerol (4.28 g, 0.0045 mol) was dissolved in aq. ethanol (85%) (40 mL) and hydrazine mono-hydrate (2.71 g, 0.054 mol) was added. The reaction mixture was stirred for 6h at 44°C. After evaporation under a stream of nitrogen, the mixture was purified by silica gel chromatography using a gradient of chloroform/methanol to give 1,2-distearoyl-3-*O*-β-D-glucosyl-(*R*/S)-glycerol (2.93 g, 0.0037 mol, 81%). Spectral data were identical to the literature; HRESIMS 809.6123 [M+Na]^+^ (calcd for C_45_H_86_O_10_Na, 809.6119 ).

**1,2-distearoyl-3-*O*-[(6’-O-trityl)-β-D-glucosyl]-(*R*/S)-glycerol**: 2-distearoyl-3-*O*-β-D-glucosyl-(*R*/S)-glycerol (2.90 g, 0.0037 mol) were treated with 1.6 equiv trityl chloride (1.64 g, 0.0059 mol) and 0.4 equiv DMAP (0.175 g, 0.0015 mol) in dry pyridine (25 mL). The reaction mixture was stirred for 3 h at 60 °C and then the organic solvent was evaporated under a stream of nitrogen. The residue was purified by silica gel chromatography using a chloroform/methanol gradient to give 1,2-distearoyl-3-*O*-[(6’-O-trityl)-β-D-glucosyl]-(*R*/S)-glycerol (2.38 g, 0.0023 mol, 77%) as pale yellow oil; ^1^H-NMR (400 MHz, CDCl_3_): δ 7.52-7.15 (15H, m, trityl portion), 5.22 (1H, m, H-2), 4.33 (1H, m, H-1a), 4.32 (1H, d, *J*= 7.0 Hz, H-1′), 4.09 (1H, m, H-1b), 4.02 (1H, m, H-3a), 3.71 (1H, m, H-3b), 3.42 (1H, m, H-3’), 3.41 (1H, H-4’), 3.40 (1H, m, H-2’), 3.40 (1H, m, H-6’a), 3.29 (1H, m, H-6’b), 2.11 (2H, t, *J*= 7.9 Hz, α-methylene), 2.09 (2H, t, *J*= 7.8 Hz, α-methylene), 1.51 (4H, m, β-methylene), 1.28-1.15 (acyl chain), 0.83 (6H, t, *J*= 7.0 Hz, 2 CH_3_); HRESIMS *m/z* 1051.7204 [M+Na]^+^ (calcd for C_64_H_10_O_10_Na, 1051.7201).

**1,2-distearoyl-3-*O*-[(2’,3’,4’-tri-acetyl-6’-O-trityl)-β-D-glucosyl]-(*R*/S)-glycerol**:1,2-distearoyl-3-*O*-[(6’-O-trityl)-β-D-glucosyl]-(*R*/S)-glycerol (2.38 g, 0.0023 mol) was dissolved in pyridine (20 mL) and acetic anhydride (10 mL). The reaction mixture was stirred for 3 h at room temperature and then extracted with distilled water and chloroform. The resulting organic phase was evaporated under reduced pressure and purified by silica gel chromatography using a light petroleum ether/diethyl ether gradient to give 1,2-distearoyl-3-*O*-[(2’,3’,4’-tri-acetyl-6’-O-trityl)-β-D-glucosyl]-(*R*/S)-glycerol (2.42 g, 0.0021 mol, 90%). ^1^H-NMR (400 MHz, CDCl_3_): δ 7.47-7.18 (15H, m, trityl portion), 5.23 (1H, m, H-2), 5.15 (1H, m, H-4’), 5.14 (1H, m, H-3’), 5.05 (1H, m, H-2’), 4.54 (1H, d, *J*= 7.7 Hz, H-1’), 4.34 (1H, dd, *J*= 4.1, 12.0 Hz, H-1a), 4.13 (1H, dd, *J*= 5.9, 12.0 Hz, H-1b), 4.03 (1H, dd, *J*= 4.9, 11.1 Hz, H-3a), 3.74 (1H, dd, *J*= 5.1, 11.1 Hz, H-3b), 3.55 (1H, m, H-5’), 3.27 (1H, bd, *J*=10.2 Hz, H-6’a), 3.09 (1H, dd, *J*=2.3, 10.2, H-6’b), 2.31 (2H, t, *J*= 7.5 Hz, α-methylene), 2.16 (2H, t, *J*= 7.8 Hz, α-methylene), 1.54 (4H, m, β-methylene), 1.28-1.15 (acyl chain), 0.86 (6H, t, *J*= 7.0 Hz, 2CH_3_); HRESIMS *m/z* 1177.7507 [M+Na]^+^ (calcd for C_70_H_106_O_13_Na, 1177.7505).

**1,2-distearoyl-3-*O*-[(2’,3’,4’-tri-acetyl)-β-D-glucosyl]-(*R*/S)-glycerol**: 1,2-distearoyl-3-*O*-[(2’,3’,4’-tri-acetyl-6’-O-trityl)-β-D-glucosyl]-(*R*/S)-glycerol (1.24 g, 0.0011 mol) was dissolved in iodine methanol solution (25 mL, 1%). After stirring for 6 h at 60 °C, the mixture was concentrated and purified by silica gel chromatography using a gradient of petroleum ether/diethyl ether to give 1,2-distearoyl-3-*O*-[(2’,3’,4’-tri-acetyl)-β-D-glucosyl]-(*R*/S)-glycerol (0.74 g, 0.00081 mol, 74%) as a pale yellow oil; ^1^H-NMR (400 MHz, CDCl_3_): δ 5.31-5.28 (2H, overlapped, H-2, H-3’), 5.08-5.00 (2H, m, H-2’, H-4’), 4.61 (1H, d, *J*= 7.8 Hz, H-1’), 4.36 (1H, m, H-1a), 4.16 (1H, m, H-1b), 3.92 (1H, m, H-3a), 3.78 (1H, m, H-3b), 3.74 (1H, m, H-6’a), 3.60 (1H, m, H-6’b), 3.54 (1H, m, H-5’), 2.41-2.29 (4H, m, α-methylene), 2.10-2.01 (9H, s, 3 OAc), 1.62-1.52 (4H, m, β-methylene), 1.33-1.15 (acyl chain), 0.90 (6H, t, *J*= 7.0 Hz, 2CH_3_); HRESIMS *m/z* 935.6399 [M+Na]^+^ (calcd for C_51_H_92_O_13_Na, 935.6403).

**1,2-distearoyl-3-*O*-[(2’,3’,4’-tri-acetyl-6’-tosyl)-β-D-glucosyl]-(*R*/S)-glycerol**: 1,2-distearoyl-3-*O*-[(2’,3’,4’-tri-acetyl)-β-D-glucosyl]-(*R*/S)-glycerol (0.74 g, 0.00081 mol) was dissolved in anhydrous pyridine (12 mL). *p*-Tosylchloride (1.69 g, 0.0089 mol) and DMAP (0.11 g, 0.00089 mol) were added at 0 °C under argon and the reaction mixture was stirred overnight at room temperature. After evaporation under a stream of nitrogen, the mixture was purified by silica gel chromatography using a gradient of petroleum ether/diethyl ether to give 1,2-distearoyl-3-*O*-[(2’,3’,4’-tri-acetyl-6’-tosyl)-β-D-glucosyl]-(*R*/S)-glycerol (0.70 g, 0.00065 mol, 80%) as a pale yellow oil; ^1^H-NMR (400 MHz, CDCl_3_): δ 7.77 (2H, d, *J*= 8.3 Hz, aromatic methynes), 7.34 (2H, d, *J*= 8.3 Hz, aromatic methynes), 5.18-5.13 (2H, m, H-2, H-3′), 4.96-4.89 (2H, m, H-2’, H-4’), 4.48 (1H, d, *J*= 7.9 Hz, H-1′), 4.26 (1H, m, H-1a), 4.08-4.01 (3H, overlapped, H-1b, H_2_-6′a), 3.87 (1H, dd, *J*=5.1, 10.8 Hz, H-3a), 3.61 (1H, dd, J= 5.4, 10.8 Hz, H-3b), 2.44 (3H, s, aromatic methyl), 2.31-2.28 (4H, m, α-methylene), 2.04-1.97 (9H, s, 3 OAc), 1.60-1.54 (4H, m, β-methylene), 1.39-1.23 (acyl chain), 0.91-0.87 (6H, overlapped, 2CH_3_); HRESIMS *m/z* 1089.6501 [M+Na]^+^ (calcd for C_58_H_98_O_15_NaS, 1089.6505).

**1,2-distearoyl-3-*O*-[(2’,3’,4’-tri-acetyl-6’-thioacetyl)-β-D-glucosyl]-(*R*/S)-glycerol**: 1,2-distearoyl-3-*O*-[(2’,3’,4’-tri-acetyl-6’-tosyl)-β-D-glucosyl]-(*R*/S)-glycerol (0.70 g, 0.00065 mol) was dissolved in 2-butanone (30 mL) and potassium thioacetate (0.19 g, 0.00163 mol). The reaction mixture was stirred at 80°C for 2.5 h and then the solvent was evaporated at reduced pressure. The resulting material was purified by silica gel chromatography using a light petroleum ether/diethyl ether gradient to give 1,2-distearoyl-3-*O*-[(2’,3’,4’-tri-acetyl-6’-thioacetyl)-β-D-glucosyl]-(*R*/S)-glycerol (0.53 g, 0.00055 mol, 84%) as a colourless oil; ^1^H-NMR (400 MHz, CDCl_3_): δ 5.18-5.14 (2H, m, H-2, H-3′), 4.96-4.89 (2H, m, H-2’, H-4’), 4.50 (1H, d, *J*= 8.0 Hz, H-1′), 4.28 (1H, dd, *J*= 4.1, 11.8 Hz, H-1a), 4.08 (1H, dd, J= 5.7, 11.8 Hz, H-1b), 3.91 (1H, dd, *J*=4.5, 11.1 Hz, H-3a), 3.65 (1H, dd, J= 5.4, 11.1 Hz, H-3b), 3.62 (1H, m, H-5’), 3.24 (1H, bd, *J*= 11.4 Hz, H-6’a), 3.05 (1H, dd, *J*= 2.4 Hz, 11.4 Hz), 2.33-2.29 (4H, m, α-methylene), 2.13-1.98 (9H, s, 3 OAc), 1.64-1.57 (4H, m, β-methylene), 1.32-1.23 (acyl chain), 0.91-0.87 (6H, overlapped, 2CH_3_); HRESIMS *m/z* 993.6322 [M+Na]^+^ (calcd for C_53_H_94_O_13_NaS, 993.6320).

**1,2-distearoyl-3-*O*-[(2’,3’,4’-tri-acetyl-)-β-D-sulfoquinovosyl]-(*R*/S)-glycerol**: 1,2-distearoyl-3-*O*-[(2’,3’,4’-tri-acetyl-6’-thioacetyl)-β-D-glucosyl]-(*R*/S)-glycerol (0.53 g, 0.00055 mol) was dissolved in potassium acetate (0.265 g, 0.00267 mol), 34% (w/v) H_2_O_2_ (1.33 mL) and acetic acid (15.7 mL). The reaction mixture was stirred overnight at 40 °C. After evaporation under a stream of nitrogen, the oily residue was purified by silica gel chromatography using a gradient of chloroform/methanol to give 1,2-distearoyl-3-*O*-[(2’,3’,4’-tri-acetyl-)-β-D-sulfoquinovosyl]-(*R*/S)-glycerol (0.41 g, 0.00040 mol, 60%) as a colourless oil; ^1^H-NMR (400 MHz, CDCl_3_): δ 5.27 (1H. m, H-2), 5.18 (1H, dd, 8.9, 8.9 Hz, H-3), 5.08-4.99 (2H, m, H-2’, H-4′), 4.68 (1H, d, 7.3 Hz, H-1’), 4.31 (1H, dd, *J*= 5.1, 11.1 Hz, H-1a), 4.15-4.06 (3H, overlapped, H-1b, H-3a, H-5’), 3.75 (1H, m, H-3b), 3.20 (2H, overlapped, H_2_-6’), 2.33-2.28 (4H, m, α-methylene), 2.06-1.98 (9H, s, 3 OAc), 1.64-1.55 (4H, m, β-methylene), 1.34-1.22 (acyl chain), 0.91-0.88 (6H, overlapped, 2CH_3_); HRESIMS *m/z* 975.6108 [M-K]^-^ (calcd for C_51_H_91_O_15_S^-^, 975.6111).

**1,2-distearoyl-3-*O*-β-D-sulfoquinovosy]-(*R*/S)-glycerol** (**6**): 1,2-distearoyl-3-*O*-[(2’,3’,4’-tri-acetyl-)-β-D-sulfoquinovosyl]-(*R*/S)-glycerol (0.40 g, 0.00040 mol) was dissolved in aq. ethanol (85%) (30 mL), hydrazine monohydrate (0.16 g, 0.00336 mol) was added, and the reaction mixture was stirred for 3h at 44°C. After evaporation under a stream of nitrogen, the mixture was purified by silica gel chromatography using a gradient of chloroform/methanol to give 1,2-distearoyl-3-*O*-β-D-sulfoquinovosy]-(*R*/S)-glycerol (**6**) (0.26 g, 0.00030 mol, 70%) as a white solid, m.p. 88-92°C; R*_f_* (chloroform/methanol 7:3) = 0.15; IR (liquid film) v_max_ 3400, 2940, 2862, 1750, 1351, 1343 cm**^-^**^1^; ^1^H-NMR (400 MHz, CDCl_3_/CD_3_OD 1/1): ^1^H-NMR (400 MHz, CDCl_3_/CD_3_OD 1/1): δ 5.29 (1H, m, H-2), 4.47 (1H, m, H-1a), 4.34 and 4.32 (each for 1H, d, 7.8 Hz, H-1’), 4.19 (1H, m, H-1b), 4.13-4.03 (1H, m, H-1a), 3.79-3.75 (2H, m, H-3b, H-5’), 3.42 (1H, m, H-3’), 3.32 (1H, m, H-6’a), 3.26 (1H, m, H-2’), 3.14 (1H, m, H-4’), 2.98 (1H, m, H-6’b), 2.43-2.35 (4H, m, α-methylene), 1.69-1.58 (4H, m, β-methylene), 1.43-1.29 (acyl chain), 0.94 (6H, overlapped, 2CH_3_).
